# Patients With Lower Limb Deformity Report Worse Quality of Life Than Control Subjects Regardless of Degree of Deformity

**DOI:** 10.5435/JAAOSGlobal-D-21-00182

**Published:** 2021-08-10

**Authors:** Madison R. Heath, Tom J. Shin, Rena Mehta, Peter S. Principe, Alexandra T. Mackie, Austin Fragomen, S. Robert Rozbruch, Peter D. Fabricant

**Affiliations:** From the Hospital for Special Surgery, New York, NY.

## Abstract

The purpose of this study was to determine how Limb Deformity Modified Scoliosis Research Society (LD-SRS) scores differ between patients with different types and degrees of limb deformity compared with control subjects.

**Methods:**

Patients who were >17 years and scheduled for reconstructive surgery for limb lengthening or angular deformity with internal and/or external fixation and healthy control subjects were prospectively enrolled. Patients completed the LD-SRS preoperatively. Mechanical axis deviation (MAD) and leg length discrepancy (LLD) were recorded preoperatively. Participants were stratified into five groups based on their diagnosis. ANOVA was used to test for associations between LD-SRS scores and diagnosis as well as mechanical axis deviation.

**Results:**

Patients with LLD, angular deformity, or combined LLD and angular deformity reported significantly worse scores than control subjects in LD-SRS Function/Activity, Pain, Self-Image/Appearance, and total score (*P* < 0.001 for all). Patients with short stature reported significantly worse LD-SRS Self-Image/Appearance (*P* < 0.001) and total score compared with control subjects (*P* = 0.015). There was a significant correlation between LLD and LD-SRS Self-Image/Appearance in the LLD and angular deformity group (r = −0.359, *P* = 0.043).

**Discussion:**

Although LD-SRS scores were worse in patients with limb deformity compared to controls, LD-SRS scores were not related to the degree of deformity in most patients, indicating that patient self-perception may be a construct unrelated to objective radiographic or clinical findings.

Lower limb deformity can have a notable effect on quality of life.^1-8^ Previous studies have reported that patients with leg length discrepancy (LLD) or angular deformity (ie, genu varum or valgum) frequently report functional limitations when compared with healthy control subject groups.^2,4,6,7^ In addition, patients with LLD, angular deformity, or short stature also report worse mental health and self-image.^5,6,8^ One study by Montpetit et al^8^ found that adolescent patients with lower limb deformities reported markedly lower scores in physical health, psychosocial health, and emotional functioning than the general population before surgical intervention. However, with recent advances in angular deformity correction and limb lengthening, surgical intervention can result in improved patient-reported and clinical outcomes.^6,9^

One important indication for surgical intervention in lower limb deformity is its effect on the patient's quality of life. To assess this effect, patient-reported outcome measures (PROMs) are often used. To be useful and valid in the clinical setting, PROMs need to be multidimensional, addressing physical health and subjective perceptions of the patients.^10^ A modified version of the Scoliosis Research Society (SRS) outcome measure, the Limb Deformity Modified SRS (LD-SRS), is currently the only limb deformity-specific patient-reported outcome measure, which was designed to capture patient perceptions of their physical function, pain, mental health, and self-image.^11^ However, differences between patients with different types and degrees of lower limb deformities using the LD-SRS have not yet been established.

Before surgical intervention, it is important to understand how lower limb deformity affects patients' perceptions of their quality of life. The purpose of this study was to determine how LD-SRS scores differ between patients with LLD, angular deformity, short stature, and healthy control subjects. In addition, this study aimed to investigate if and how different degrees or severities of deformity affect patient-reported quality of life. The investigators hypothesized that patients with LLD, angular deformity and short stature would have worse LD-SRS scores than healthy control subjects.

## Methods

After obtaining IRB approval, patients scheduled for a limb deformity surgery at the study institution were prospectively enrolled. All included patients were at least 18 years of age and were scheduled for complex reconstructive surgery for limb lengthening or angular deformity with internal and/or external fixation. In addition, 30 healthy participants who were at least 18 years of age with no history of lower extremity surgery were enrolled prospectively through convenience sampling.

All participants completed the LD-SRS and a suite of PROMIS domains (Global Mental Health, Global Physical Health, Pain Interference, Physical Function, and Pain Intensity). Similar to the SRS instrument, the LD-SRS has four domains including Function/Activity, Pain, Mental Health, and Self Image/Appearance. Each domain is scored from 1 to 5, with a higher score indicating better quality of life (eg, a high LD-SRS Pain score indicates low pain levels). The total LD-SRS score is an average of the four domains, and a higher score indicates overall better outcomes. The minimum clinically important difference for the LD-SRS total score is 0.3.^11^ The PROMIS domains are normalized to a mean of 50 with an SD of 10. Higher scores indicate more of the domain being measured (eg, a high PROMIS Pain Interference score indicates a high level of pain that interferes with daily life).

Participants were stratified into five groups based on their underlying conditions as follows: (1) short stature, (2) LLD, (3) angular deformity, (4) LLD with angular deformity, and (5) normal control subjects. Surgery for short stature was indicated for patients who presented at or below the fifth height percentile for their age after psychological assessment to rule out underlying psychological conditions (eg, body dysmorphic disorder). For all the noncontrol patient groups, mechanical axis deviation (MAD) and LLD were recorded from the medical chart. The greatest MAD was calculated between each leg for the patients, and this value was used in statistical analyses. The indication for surgery for LLD was a difference of greater than 18 mm between leg lengths, and for angular deformity, it was a deformity of greater than 5° with symptoms.

Descriptive statistics were reported for all demographic variables. Frequencies and percentages were reported for categorical variables, and means and SDs were reported for continuous variables. One-way ANOVA with Tukey post hoc pairwise analysis was used to determine statistically significant differences between groups of patients. Within the LLD group and the LLD with angular deformity group, Pearson correlation coefficients were calculated for LLD with the PROMs. In addition, within the angular deformity and angular deformity with LLD groups, Pearson correlations were calculated for the greatest MAD in each patient with the PROMs. All tests were two-tailed with significance threshold set at *P* = 0.05.

## Results

A total of 240 participants were included in this study, including 18 patients with short stature, 49 patients with an LLD, 94 patients with an angular deformity, 49 patients with a combined LLD and angular deformity, and 30 normal control subjects. Patients reported bilateral deformities in two LLD cases, 13 angular deformity cases, and two combined LLD and angular deformity cases. Demographic characteristics are provided in Table 1.

**Table 1:** Demographic Characteristics of Included Study Participants

Demographic Characteristic	N (%)	M (SD)
Sex		
Male	132 (55)	—
Female	108 (45)	—
Race		
White	149 (62)	—
African American	24 (10)	—
Asian	27 (11)	—
Other	39 (16)	—
Declined	1 (1)	—
Ethnicity		
Hispanic	23 (10)	—
Non-Hispanic	212 (88)	—
Declined	5 (2)	—
Age	—	36.5 (16.7)
BMI	—	26.0 (6.6)

**Figure 1:**
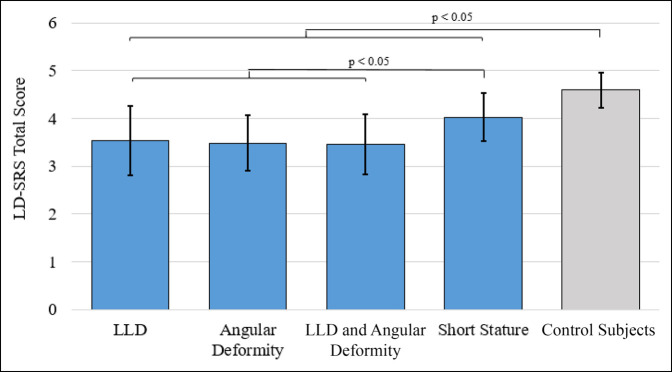
Graph showing mean LD-SRS total scores for each patient with leg length discrepancy (LLD), angular deformity, LLD with combined angular deformity, short stature, and control subjects. Error bars represent 1 SD of the mean. Notable differences between groups are denoted with brackets.

Patients with LLD, angular deformity, or LLD with combined angular deformity reported significantly worse scores than control subjects in LD-SRS Function/Activity, Pain, Self-Image/Appearance, and total score (*P* < 0.001 for all domains; Table 2). Patients with short stature reported significantly worse scores than control subjects in LD-SRS Self-Image/Appearance (*P* < 0.001) and total score (*P* = 0.015) but were not significantly different in any of the other domains (Function/Activity, Pain, Mental Health). All significant differences between groups on the LD-SRS total are shown in Figure 1. The mean LD-SRS total score for all patient groups was lower than the control subject group, and exceeded the minimum clinically important difference of 0.3. There were no notable differences between LLD, angular deformity, and LLD with angular deformity groups for any LD-SRS domains. Patients with short stature reported significantly better scores than other patient groups for LD-SRS Function/Activity, Pain, and total score (*P* < 0.01 for all) but were not significantly different from other patient groups in Self-Image/Appearance. Patients with LLD with angular deformity reported significantly worse Mental Health outcomes than control subjects (*P* = 0.021), but there were no other significant differences between groups in the Mental Health domain.

**Table 2:** Limb Deformity-SRS (LD-SRS) Scores by the Study Group

LD-SRS Domain	LLD	Angular Deformity	LLD and Angular Deformity	Short Stature	Control Subjects
	M (SD)	M (SD)	M (SD)	M (SD)	M (SD)
Function/Activity	3.34 (0.93)^a^	3.44 (0.78)^a^	3.42 (0.87)^a^	4.16 (0.62)	4.47 (0.42)
Pain	3.65 (0.93)^a^	3.57 (0.81)^a^	3.61 (0.97)^a^	4.81 (0.35)	4.84 (0.26)
Mental health	4.00 (0.82)	3.91 (0.74)	3.76 (0.75)^a^	3.94 (0.81)	4.31 (0.74)
Self-Image/Appearance	3.15 (0.83)^a^	3.00 (0.78)^a^	3.07 (0.71)^a^	3.19 (0.79)^a^	4.73 (0.43)
Total	3.53 (0.73)^a^	3.48 (0.58)^a^	3.46 (0.63)^a^	4.02 (0.50)^a^	4.59 (0.37)

LD-SRS = Limb Deformity-SRS, LLD = leg length discrepancy; SRS = Scoliosis Research Society

a Indicates a significant difference (*P* < 0.05) from the control subject group.

In the PROMIS domains, patients with LLD, angular deformity, or LLD with angular deformity reported significantly worse scores than control subjects and patients with short stature in Pain Interference, Physical Function, Pain Intensity, and Global Physical Health (*P* < 0.001 for all; Table 3). There were no significant differences between LLD, angular deformity, and LLD with angular deformity groups in any of the PROMIS domains. There were also no significant differences between patients with short stature and control subjects in any of the PROMIS domains. Finally, patients with LLD reported significantly worse Global Mental Health than control subjects (*P* = 0.020), but there were no other significant differences between groups in that domain.

**Table 3:** PROMIS Scores by the Study Group

PROMIS Domain	LLD		Angular Deformity		LLD and Angular Deformity		Short Stature		Control Subjects	
	n	M (SD)	n	M (SD)	n	M (SD)	n	M (SD)	n	M (SD)
Physical Function	47	41.7 (12.6)^a^	87	42.5 (9.91)^a^	44	42.2 (9.34)^a^	18	59.0 (11.5)	30	60.3 (7.4)
Pain Interference	31	58.7 (9.8)^a^	76	57.9 (7.7)^a^	41	55.5 (9.4)^a^	11	42.2 (8.1)	30	42.4 (5.6)
Pain Intensity	44	44.2 (10.2)^a^	85	46.9 (8.6)^a^	44	45.9 (9.2)^a^	18	33.5 (5.3)	30	33.1 (4.2)
Global Mental Health	32	48.3 (9.1)^a^	78	52.5 (8.6)	41	49.7 (9.8)^a^	12	50.9 (10.6)	30	55.3 (7.8)
Global Physical Health	32	43.1 (11.3)^a^	78	45.8 (8.0)^a^	41	44.8 (7.7)^a^	12	58.5 (8.8)	30	57.9 (7.1)

LLD = leg length discrepancy

a Indicates a significant difference (*P* < 0.05) from the control subject group.

When assessing the degree of deformity within patient groups, no significant correlations were found between LLD and LD-SRS scores in the LLD group, but there was a significant correlation between LLD and LD-SRS Self-Image/Appearance in the LLD with combined angular deformity group (r = −0.359, *P* = 0.043). In addition, there was no correlation between MAD and LD-SRS scores in the angular deformity group or the LLD with angular deformity group.

## Discussion

The purpose of this study was to characterize baseline/preoperative PROMIS and LD-SRS scores in patients with lower limb deformity compared with healthy control subjects. To this end, this study found that patients with LLD, angular deformity, or both reported worse physical function/activity, pain, and self-image/appearance than healthy control subjects based on PROMIS and LD-SRS outcome scores. Previous studies have also found functional limitations, pain, and lower self-image in patients with LLD or angular deformity.^2,4,6-8^ These findings are important for understanding the deficits caused by these lower limb deformities and how to assess them in a clinical setting as a possible indication for surgery.

Another important finding of this study was the self-image/appearance outcome in patients with short stature. Although these patients did not display the functional limitations or pain that other patients with lower limb deformity reported, patients with short stature reported low self-image related to their lower limbs, similar to that of patients with LLD or angular deformity. Previous studies have shown that short stature can have a notable effect on psychosocial health.^5^ Limb lengthening complications are not uncommon,^12^ but many patients report higher self-esteem and overall satisfaction after the procedure.^13,14^ It is important to determine a patient's perspective when considering surgical intervention for short stature. In addition, the LD-SRS was able to detect this difference in self-image between patients with short stature and control subjects, whereas the other PROMIS domains were not. This indicates that PROMs specific to lower limb deformity that assess self-image may be essential in determining a patient's perception.

Another notable finding from this study was that, with the exception of self-image in patients with LLD with angular deformity, degree/severity of deformity was seemingly not related to patients' perception of their self-image/appearance, function/activity, pain, or mental health. Previous studies of patients with adolescent idiopathic scoliosis (AIS) found that larger Cobb angles preoperatively were associated with worse pain, function, activity, and self-image.^15,16^ Another study in children found worse psychosocial outcomes with increasing LLD.^17^ However, the LD-SRS was designed to capture patient self-perceptions rather than objective clinical findings,^11^ which may explain why the magnitude of LLD or angular deformity was not associated with LD-SRS scores in most patients.

One of the strengths of this study includes the use of a lower limb deformity-specific validated patient-reported outcome measure. While more general Patient-Reported Outcomes Measurement Information System (PROMIS) scales did capture notable differences in outcomes between the patients with limb deformity and control subjects, the LD-SRS also reported differences in self-image/appearance and used fewer overall questions within the single questionnaire. There is currently no PROMIS domain for assessing deformity-related self-image. Therefore, the LD-SRS is an optimal way to assess presurgical lower limb deformity patient perceptions with low burden to the patient.

In addition to its strengths, this study had several limitations. Because this was a retrospective analysis of prospectively collected data, no a priori power analysis was done. In addition, some patient groups had few participants, so some analyses may have been underpowered. Although this does not weaken the positive findings of this study, drawing conclusions using nonsignificant associations should be made with caution. In addition, several PROMIS forms were incomplete for the included participants, reducing the sample size for PROMIS analyses and therefore weakening conclusions related to PROMIS scores. Another limitation was the lack of an additional self-image or appearance-related scale with which to compare the LD-SRS scores. However, no applicable self-image domain currently exists for PROMIS.^18,19^ In addition, patients were not categorized by underlying diagnosis or number of deformities. These factors might have an effect on self-perception as well. Future prospective studies with more participants should study how these diagnoses affect self-perception. Finally, the investigators selected a small convenience sample of healthy control subjects because of hospital COVID-19 restrictions on research. These control subjects may have had different demographic characteristics than the study population, which may have introduced bias of unclear clinical relevance. Future studies could investigate this further with more closely matched control subjects.

In conclusion, this study found notable differences in physical function, pain, and self-image between patients with lower limb deformities and healthy control subjects. This study further validates the LD-SRS as an objective and quantitative measure of patient perception and quality of life in patients with lower limb deformity. Radiographic measurements of degree/severity of deformity were not related to LD-SRS scores on average, indicating that patient self-perception may be unrelated to objective radiographic or clinical findings.
